# The Rising Triad of Cesarean Scar Pregnancy, Placenta Percreta, and Uterine Rupture: A Case Report and Comprehensive Review of the Literature

**DOI:** 10.1155/2018/8797643

**Published:** 2018-06-07

**Authors:** Nikolina Docheva, Emily D. Slutsky, Nicolette Borella, Renee Mason, James W. Van Hook, Sonyoung Seo-Patel

**Affiliations:** ^1^Department of Obstetrics and Gynecology, University of Toledo, Toledo, Ohio, USA; ^2^Mercyhurst University, Department of Biology, Eerie, Pennsylvania, USA; ^3^Promedica Physicians Obstetrics-Gynecology, Maumee, Ohio, USA

## Abstract

As the rate of cesarean sections continues to rapidly rise, knowledge of diagnosis and management of cesarean scar pregnancies (CSPs) is becoming increasingly more relevant. CSPs rest on the continuum of placental abnormalities which include morbidly adherent placenta (accreta, increta, and percreta). A CSP poses a clinical challenge which may have significant fetal and maternal morbidity. At this point, no clear management guidelines and recommendations exist.* Herein* we describe the case of a second trimester CSP with rapid diagnosis and management in a tertiary care center. The case underscores the need for well-coordinated mobilization of resources and a multidisciplinary approach. A review of the literature is performed and deficits in universal management principles are underscored.

## 1. Introduction

Over the last two decades there has been a rapid growth of the number of cesarean sections performed and, in 2014, 1 in 3 women who gave birth in the USA did so by cesarean section [1]. This trend has been largely attributed to the rise of the “primary cesarean section,” with a corresponding decrease in operative vaginal deliveries [1, 2]. Yet, no significant decrease in maternal and neonatal morbidity and mortality has been observed [3]. Even though vaginal delivery after a cesarean section is endorsed by ACOG for appropriate candidates, the rate of repeat cesarean deliveries is now close to 91% [2].

A cesarean section is not a benign procedure and is associated with an increased risk of maternal and fetal morbidity and mortality [4]. A rare complication of cesarean section is a “cesarean scar pregnancy” (CSP), which is also known as “cesarean ectopic pregnancy,” and “cesarean delivery scar pregnancy.”

## 2. Case Presentation

A 34-year-old Gravida 11 Para 3073 at 16 weeks and 1 day gestation presented to the emergency room of an outside hospital with a 2-day history of progressively worsening nausea, vomiting, and diarrhea, exacerbated by eating. The pregnancy had been unremarkable. Her past medical history included endometriosis and infertility. Her past surgical history was significant for two cesarean sections and left salpingo-oophorectomy secondary to an ectopic pregnancy. Physical exam elicited severe, diffuse abdominal tenderness. Fetal heart tones were taken to be in the 140s and positive fetal movement was reported. Laboratory investigations, including complete blood count, comprehensive metabolic panel, amylase, and lipase, were within normal limits. The ER physician's leading differential diagnosis was of gastrointestinal etiology. An MRI and MRCP were performed to rule out appendicitis and gallbladder disease. The MRI was notable for a large amount of intraperitoneal fluid of unknown etiology; an intrauterine fetus was visualized.

**Figure 1:**
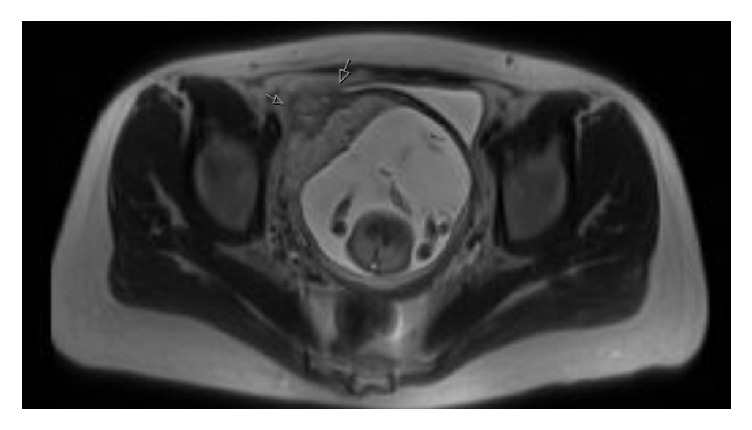
Cross-sectional MRI showing intrauterine pregnancy and CSP with suggestion of placental invasion to the bladder.

The patient continued to experience intractable pain, worse with movement and breathing, despite IV pain medication. At that point she has been at the outside facility for approximately 12 hours. The patient was transferred to our facility under the joint care of the Obstetrics/Gynecology and General Surgery teams. Upon arrival, the patient's hemodynamic status had deteriorated. She presented with tachycardia, dyspnea, chest pain, and worsening abdominal pain. Her hemoglobin had fallen from 11.7 g/dL to 7.9 g/dL. Transabdominal ultrasound imaging revealed a single intrauterine pregnancy that was positioned low in the uterus, with marked thinning of the anterior myometrium at the site of the pregnancy, and significant hemoperitoneum. Fetal heart tones were steady in the 140s. The MRI images were reevaluated prior to surgery (see Figure 1).

At this point, the patient was taken for emergency laparotomy and the staff Gynecologic Oncologist was consulted. The patient underwent a modified radical hysterectomy with right ureteral lysis and cystotomy with bladder repair. The intraoperative findings were consistent for a placenta percreta and uterine rupture with a 2 x 1 cm defect in the right lower uterine segment. There were significant intra-abdominal blood and evidence of invasion of the placenta into the posterior aspect of the bladder. Total estimated blood loss for the surgery was 3,150 mL. The patient received 900 mL of cell saver and 1 unit packed red blood cells (PRBC) intraoperatively.

The patient was admitted to the ICU following surgery. She was transferred out of the unit on postoperative day 1. Two more units of PRBC were transfused over the course of the postoperative period. She was discharged on postoperative day 4 after having met her postoperative milestones. Due to the cystotomy, she was discharged with Foley urinary catheter in place for a minimum of 7 days with cystogram scheduled prior to removal. Patient was referred for grief counseling.

**Figure 2:**
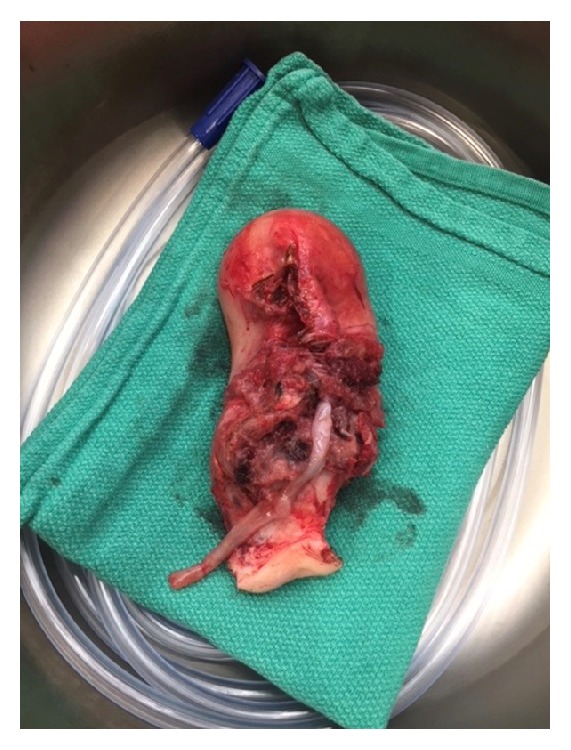
Gross specimen showing uterine rupture.

Pathologic examination of the uterus included placenta percreta with uterine rupture (see Figure 2 for gross specimen). There was absence of decidua identified in the lower uterine segment in the area of the uterine rupture.

## 3. Discussion

A CSP is not an “ectopic” pregnancy and instead involves the implantation (part or whole) of the gestation and the placenta into the niche (dehiscence at the hysterotomy site) or scar of the prior cesarean section [5, 6]. The estimated incidence of CSP is reported to range from 1 in 1,800 to 1 in 2,500 of all cesarean deliveries performed [6, 7].

It has been proposed that, in CSPs, invasion of the conceptus occurs through a defect or microscopic dehiscence in the scar or niche. This is believed to be secondary to the poor vascularization with fibrosis of the lower uterine segment [7].

Another complication of cesarean section and CSP is the morbidly adherent placenta (accreta, increta, and percreta). As more cases are now reported in the literature, it is believed that CSP and a morbidly adherent placenta are on a continuum spectrum of implantation abnormalities starting with CSP and progressing to deeper placental invasion as the gestation advances. This is supported by evidence which shows that these entities are indistinguishable histopathologically [8].

CSP is a clinical challenge as it can manifest broadly in two ways: (1) during the time of an ultrasound examination in a patient with a prior cesarean section and (2) as an acute emergency as described in this case report [4, 6, 7]. Due to its rarity, however, the natural history of CSP has proven difficult to study and most of what we know has been based on case reports and case series. Well-known complications from CSP include morbidly adherent placenta, uterine rupture, hemorrhage, preterm labor, fetal demise, arteriovenous malformation, need for uterine artery embolization, hysterectomy, and even maternal death [6, 7, 9].

Rotas et al. in their review of cases of CSPs, showed that 36.8% (21/57) of patients were asymptomatic, 38.6% (22/57) had painless vaginal bleeding, 15.8% (9/57) had abdominal pain with bleeding, and 8.8% (5/57) had only abdominal pain [7]. This heterogenous presentation of patients with CSPs can be attributed to the continuum of the condition, the gestational age, the type of CSP, and implantation in the scar versus implantation in the niche. Patients with CSP implanted in the fully healed scar have a better outcome than those with CSP implanted in the niche [5].

Diagnosis of CSP is based on combination of patient's history, clinical manifestations, and imaging. Accurate and prompt diagnosis of the condition is crucial as it can be life-threatening [6, 7]. Transvaginal ultrasound is the main imaging modality for the diagnosis of CSP with a sensitivity of 84.6% (95% CI 0.763-0.905) [7]. Color Doppler imaging, 3-dimensional power Doppler ultrasonography, and 3-dimensional vocal imaging systems have also been used to evaluate the flow, resistance, and pulsatility indices of the vasculature at or in the area of the hysterotomy [6, 7]. Other diagnostic imaging modalities such as MRI can be used as an adjuvant to ultrasound as well as aid in preparation for surgery and intraoperative orientation. In addition, endoscopic modalities such as cystoscopy can be used to rule out bladder invasion and hysteroscopy can be used for improved visualization. In our case, the patient had two ultrasounds early in the pregnancy, one to confirm viability and the second for nuchal translucency. However, in both of those the lower uterine segment was not evaluated. When she presented to the outside hospital, her initial symptoms were worrisome for a gastrointestinal etiology of pain. Recommendation from Radiology included performing MRCP and MRI, thereby avoiding CT scan. The MRI revealed free intraperitoneal fluid. The patient was then transferred to our hospital, a tertiary care center, where ultrasound was used to reevaluate the pain of the patient and monitor the fetal status. The transabdominal imaging confirmed hemoperitoneum and suspected uterine rupture. The patient was promptly taken to the operating room for an exploratory laparotomy. The MRI was beneficial to the surgical team because it allowed them to perform placental mapping and evaluate the invasion of the bladder. Ultrasound remains the first-line imaging modality, as it provides results in timely fashion, has high sensitivity, is accessible, and is highly cost-effective.

Currently, there is no consensus on the treatment and management of CSP. It is clear that early diagnosis and treatment are ideal for minimizing complications and preserving fertility. There is currently no recommendation or literature which supports expectant management and thus treatment must be pursued. Currently, treatment is individualized and inconsistent as CSPs are rare and physicians are often underexperienced in the area [10].

The role for systemic methotrexate (MTX) is limited to gestational age <8 weeks, absence of fetal cardiac activity, and beta-human chorionic gonadotropin (beta-hCG) levels < 12,000 mIU/mL [11]. When criteria are met, this is considered first-line treatment. Multidose MTX treatment regimens have not been formally studied. Additionally, local MTX is associated with a success rate of 61.1% and can be applied to pregnancies with beta-HCG < 20,000 mIU/mL and a mass less than 3 cm in diameter [12].

Timor-Tritsch et al. advocated for the adjuvant use of an inflatable Foley catheter following MTX injection as prevention of hemorrhage however noted the risk of balloon expulsion within 3 days of placement [13]. The subsequent introduction of the double-balloon catheter, however, addressed balloon expulsion with better ability to tamponade bleeding. The double-balloon catheter was also used to successfully terminate the pregnancy [14].

Local embryocides, such as potassium chloride, crystalline trichosanthin with mifepristone, and hyperosmolar glucose, have been similarly used, although with a high failure rate and need for rescue hysterectomies to control hemorrhage [15–19].

Uterine artery embolization (UAE) has been studied as a conservative therapy for various gynecologic and obstetric indications such as postpartum hemorrhage and uterine fibroids. It has been used for the treatment of CSP, in combination with local MTX therapy [20, 21] or curettage [22]. Because one of the goals of CSP management is the preservation of fertility, the use of UAE has been met with hesitancy. UAE can contribute to hypomenorrhea secondary to uterine and endometrial necrosis. No large-scale studies on long-term fertility following UAE have been completed to this point.

Hysteroscopy alone can rarely successfully be used – and not many cases have been published with standalone hysteroscopic treatment [23] The criteria for successful hysteroscopic management include (1) gestational sac with or without fetal pole; (2) presence or absence of fetal heartbeat; (3) location of sac in the anterior part of the uterine isthmus; (4) an empty uterine cavity without contact with the sac; (5) a clearly visible cervical canal; and (6) absence of a defect in the myometrial tissue between the bladder and the sac [24, 25]. Bleeding can be successfully controlled with coagulation or Foley catheter tamponade [26]. Follow-up should be diligently performed with serial beta-HCG levels. Hysteroscopy has also proven useful as an adjunct when systemic methotrexate had been insufficiently effective in controlling vaginal bleeding [27].

Dilation and curettage is not only rarely successful as the sole management technique—21 cases were identified by Rotas et al. with only five as not requiring any further treatment or intervention. 23.8% or 3/21 required hysterectomy after severe hemorrhage [7]. Dilation and curettage as a treatment option results in significant complications requiring additional and more invasive measures such as hysterectomy, laparoscopy, or systemic methotrexate.

Laparoscopic removal of a CSP has been reported in a few case reports as well with precautions to minimize bleeding. Wang et al. describe a case in which successful laparoscopic evacuation of a CSP hinged on the use of bilateral uterine artery ligation before excision [28]. Additional successful laparoscopic case reports advocated the use of hysteroscopy as diagnostic confirmation [29]. Also, vasopressin may be used to minimize bleeding at the time of the procedure [30]. There have been strides in the field to begin using robotic assisted laparoscopy for the surgical excision of CSPs [31].

As we strive to improve identification and diagnosis of CSP by early trimester ultrasound and Doppler imaging, a standardized, evidence-based approach to CSP management should be clarified. The techniques described above have been successfully and unsuccessfully used in combination. The individualization of approaches is not yet clear but hinges on gestational age, hemodynamic stability, anatomical complications, and surgeon's comfort level and access to resources. The aim of minimally invasive techniques is the avoidance of the peripartum hysterectomy, which is associated with substantial risk—damage to the bladder, bowel, and ureters with potential for short-term and long-term complications [32, 33]. The psychological weight of simultaneously losing a pregnancy and a loss of future fertility can provide a huge burden to patients.

If laparotomy is indicated, the Triple-P procedure has been offered as a reasonable alternative, particularly with a morbidly adherent placenta. Chandraharan describes the surgery as follows: perioperative placental localization and delivery of the fetus via transverse uterine incision above the upper border of the placenta, pelvic devascularization, and placental nonseparation with myometrial excision and reconstruction of the uterine wall [34]. Multiple successful cases were described [35].

There are several case reports which described presentation of second trimester CSPs as uterine rupture. Common initial findings including acute abdomen, hemodynamic instability, and acute blood loss require resuscitative measures. In all cases, emergency laparotomy was performed with significant extravasation of blood within the peritoneal cavity [36–38].

A case series included the use of suction dilation and curettage in the management of early second trimester CSPs. Two of the three cases culminated in hysterectomies due to pathologically adherent placentas [39].

Patients who undergo treatment must ideally adhere to close long-term surveillance. On average, resolution of beta-hCG levels occurred over the course of 88.6 days (range: 26-177) when treatment took place with any method outside of hysterectomy and UAE [6]. Beta-hCG will initially increase, as observed when following ectopic pregnancies. One suggested mechanism for this is the resulting necrosis of trophoblastic cells and the release of stored beta-hCG within the cells [6]. Timor-Tritsch et al. also recommend the use of 3-dimensional ultrasound with power Doppler to compare the vascular density over time [6]. This has not been thoroughly studied yet.

There is a crucial need for counseling patients accurately about the risks of subsequent pregnancy in this population. Regardless of minimally invasive treatment technique, patients with previous CSP are at high risk of future uterine rupture, hemorrhage secondary to placenta implantation abnormalities, and recurrence of CSP. Out of 27 patients cited in recent literature, 19 subsequent pregnancies went to term [40]. A review of the literature by Gao et al. found that uneventful term intrauterine pregnancies occurred following all the above described modalities of treatment of CSPs, despite the risks involved [41]. The successful pregnancy rate was 87.5% with the rate of recurrence of CSP at 11.1%. Live birth rate was 62.5%. Of note, uterine defect repair did not significantly improve outcomes [41]. Timing of delivery was inconsistent; Gao et al. suggest that repeat cesarean section should occur when fetal lung maturity was confirmed, while other publications reported positive outcomes with performing term repeat cesarean sections [41, 42].

Although there have been case reports and series of CSPs carried to term [43], with even live births in patients with several recurrent CSPs [44], the overarching theme remains that perinatal and maternal risks are significant. The CSPs that do continue to term have overwhelmingly resulted in placenta percreta and hysterectomy.
